# A triclinic polymorph of 3-nitro­anilinium chloride

**DOI:** 10.1107/S1600536811029072

**Published:** 2011-07-23

**Authors:** S. Thangarasu, S. Athimoolam, S. Asath Bahadur

**Affiliations:** a Department of Physics, Kalasalingam University, Anand Nagar, Krishnan Koil 626 190, India; b Department of Physics, University College of Engineering Nagercoil, Anna University of Technology Tirunelveli, Nagercoil 629 004, India

## Abstract

The asymmetric unit of the title compound, C_6_H_7_N_2_O_2_^+^·Cl^−^, contains two independent ion pairs. A monoclinic form of the title compound with only one ion pair in the asymmetric unit has been reported previously [Ploug-Sørensen & Andersen (1986). *Acta Cryst*. C**42**, 1813–1815]. In the crystal of the title compound, the components are linked into layers parallel to (001) by inter­molecular N—H⋯Cl hydrogen bonds, with alternating hydro­philic and hydro­phobic regions.

## Related literature

For the monoclinic polymorph of the title compound, see: Ploug-Sørensen & Andersen (1986) ▶. For the applications of nitro­anilines, see: Jain *et al.* (2005 ▶); Teng & Garito (1983 ▶). For information on polymorphism, see: Davey (2003 ▶); Li *et al.* (2001 ▶); Rodríguez-Spong *et al.* (2004 ▶). For hydrogen-bond motifs, see: Etter *et al.*, (1990 ▶). 
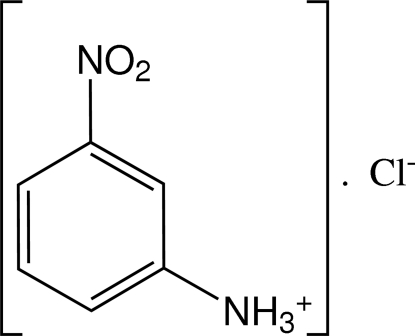


## Experimental

### 

#### Crystal data

C_6_H_7_N_2_O_2_^+^·Cl^−^
*M* *_r_* = 174.59
Triclinic, 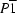

*a* = 6.9936 (8) Å
*b* = 7.8608 (9) Å
*c* = 14.6708 (16) Å
α = 87.079 (19)°
β = 81.813 (19)°
γ = 73.597 (17)°
*V* = 765.77 (15) Å^3^
*Z* = 4
Mo *K*α radiation
μ = 0.45 mm^−1^
*T* = 293 K
0.21 × 0.18 × 0.13 mm

#### Data collection

Bruker SMART APEX CCD diffractometer
5204 measured reflections
2640 independent reflections
2348 reflections with *I* > 2σ(*I*)
*R*_int_ = 0.028

#### Refinement

*R*[*F* ^2^ > 2σ(*F* ^2^)] = 0.039
*wR*(*F* ^2^) = 0.139
*S* = 1.22
2640 reflections
223 parameters
H atoms treated by a mixture of independent and constrained refinement
Δρ_max_ = 0.34 e Å^−3^
Δρ_min_ = −0.28 e Å^−3^

### 

Data collection: *SMART* (Bruker, 2001 ▶); cell refinement: *SAINT* (Bruker, 2001 ▶); data reduction: *SAINT*; program(s) used to solve structure: *SHELXTL* (Sheldrick, 2008 ▶); program(s) used to refine structure: *SHELXTL*; molecular graphics: *PLATON* (Spek, 2009 ▶); software used to prepare material for publication: *SHELXTL/PC* (Sheldrick, 2008 ▶).

## Supplementary Material

Crystal structure: contains datablock(s) global, I. DOI: 10.1107/S1600536811029072/lh5286sup1.cif

Structure factors: contains datablock(s) I. DOI: 10.1107/S1600536811029072/lh5286Isup2.hkl

Supplementary material file. DOI: 10.1107/S1600536811029072/lh5286Isup3.cml

Additional supplementary materials:  crystallographic information; 3D view; checkCIF report

## Figures and Tables

**Table 1:** Hydrogen-bond geometry (Å, °)

*D*—H⋯*A*	*D*—H	H⋯*A*	*D*⋯*A*	*D*—H⋯*A*
N12—H12*A*⋯Cl2^i^	0.93 (4)	2.28 (4)	3.164 (4)	161 (3)
N12—H12*B*⋯Cl2^ii^	0.86 (4)	2.35 (4)	3.204 (4)	170 (3)
N12—H12*C*⋯Cl1^iii^	0.85 (4)	2.68 (4)	3.445 (5)	151 (3)
N12—H12*C*⋯Cl1^iv^	0.85 (4)	2.69 (4)	3.221 (3)	123 (3)
N22—H22*A*⋯Cl1	0.86 (4)	2.33 (4)	3.174 (4)	166 (3)
N22—H22*B*⋯Cl2	0.84 (4)	2.40 (4)	3.218 (4)	165 (3)
N22—H22*C*⋯Cl1^v^	0.93 (4)	2.21 (4)	3.138 (3)	170 (3)

Symmetry codes: (i) 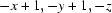; (ii) 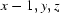; (iii) 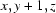; (iv) 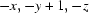; (v) 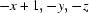.

**Fig. 1.**
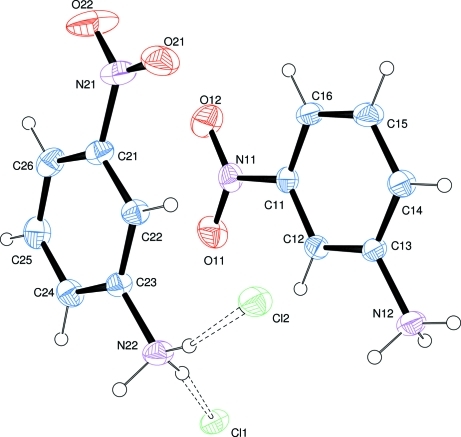
The molecular structure of the title compound with 50% displacement ellipsoids. H-bonds are shown as dashed lines.

**Fig. 2.**
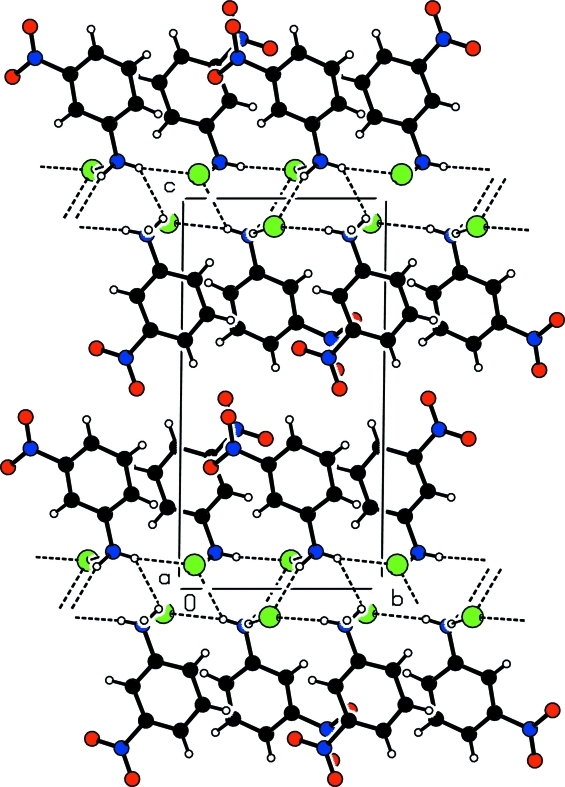
Packing diagram of the title compound viewed down the a-axis. H-bonds are shown as dashed lines.

## supplementary crystallographic information

### Comment

Nitroanilines belong to the so-called push–pull molecules due to the intramolecular charge transfer (ICT) from the –NH_2_ electron-donor group, through the phenyl ring, to the electron-acceptor –NO_2_ group. 4-Nitroaniline and 3-nitroaniline serve as the reference compounds in both, experimental and computational studies on optical nonlinearity (Teng & Garito, 1983). Also 3-Nitroaniline and its derivatives are biologically important compounds owing to the production of significant hypoglycemic as well as antihyperglycemic effects in normal and alloxan-induced diabetic rabbits (Jain *et al.*, 2005).  Organic molecules including most of pharmaceutical compounds are prone to polymorphic formation in the solid state. Variations in crystallization environment (*e.g.*, solvent, temperature, using as additives and concentration), can cause the same molecules to pack differently and form different crystal lattices or polymorphs (Davey, 2003). As a result, the physical, chemical and mechanical properties of crystals can be dramatically affected (Li *et al.*, 2001). It is now widely appreciated that the occurrence of polymorphism in molecular crystalline solids impacts on the production of fine chemical products such as pharmaceuticals, pigments and photographic couplers (Rodríguez-Spong *et al.*, 2004).

The title compound, (I), crystallizes with two crystallographically independent 3-nitroanilinium cations and two chloride anions in the asymmetric unit of the triclic unit cell, with the spacegroup *P*1. A monoclinic form with space group *P*2_1_/*c* has been previously reported by Ploug-Sørensen & Andersen (1986). In the title compound the nitro groups of the cations are twisted from the plane of the aromatic rings by 0.32 (3) and 7.1 (3)°. The protonation on the N atom of the cations are confirmed from the elongated C—N bond distances.

The crystal packing, is stabilized through intermolecular N—H···Cl interactions, as shown in Fig. 2 and hydrogen bond parameters are listed in Table 1. All ammonium H atoms of the cations are involved in the hydrogen bonds with the chloride anions. The cations and anions are connected to form *R*_4_^2^(8) ring motifs (Etter *et al.*, 1990). Overall, the components are linked into two-dimensional layers parallel to (001) by the intermolecular N—H···Cl hydrogen bonds. This type of aggregation forms alternating hydrophilic and hydrophobic regions.

### Experimental

The title compound, (I), was crystallized from an aqueous mixture of 3-nitroaniline and hydrochloric acid in the stochiometric ratio of 1:1 at room temperature, by the technique of slow evaporation.

### Refinement

The H atoms, which participate in hydrogen bonds, were located in difference Fourier maps and then refined isotropically [N—H = 0.84 (4) - 0.93 (4)Å]. H atoms bonded to C atoms were treated in a riding-model approximation, with d(C—H) = 0.93 Å and *U*_iso_(H)= 1.2 *U*_eq_(C).

### Crystal data

**Table:**

C_6_H_7_N_2_O_2_^+^·Cl^−^	*Z* = 4
*M**_r_* = 174.59	*F*(000) = 360
Triclinic, *P*1	*D*_x_ = 1.514 Mg m^−^^3^*D*_m_ = 1.49 (1) Mg m^−^^3^*D*_m_ measured by flotation technique using a liquid-mixture of xylene and bromoform
Hall symbol: -P 1	Mo *K*α radiation, λ = 0.71073 Å
*a* = 6.9936 (8) Å	Cell parameters from 2545 reflections
*b* = 7.8608 (9) Å	θ = 2.7–24.9°
*c* = 14.6708 (16) Å	µ = 0.45 mm^−^^1^
α = 87.079 (19)°	*T* = 293 K
β = 81.813 (19)°	Block, colourless
γ = 73.597 (17)°	0.21 × 0.18 × 0.13 mm
*V* = 765.77 (15) Å^3^	

### Data collection

**Table:**

Bruker SMART APEX CCD diffractometer	2348 reflections with *I* > 2σ(*I*)
Radiation source: fine-focus sealed tube	*R*_int_ = 0.028
graphite	θ_max_ = 25.0°, θ_min_ = 2.7°
ω scans	*h* = −8→8
5204 measured reflections	*k* = −9→9
2640 independent reflections	*l* = −16→17

### Refinement

**Table:**

Refinement on *F*^2^	Primary atom site location: structure-invariant direct methods
Least-squares matrix: full	Secondary atom site location: difference Fourier map
*R*[*F*^2^ > 2σ(*F*^2^)] = 0.039	Hydrogen site location: inferred from neighbouring sites
*wR*(*F*^2^) = 0.139	H atoms treated by a mixture of independent and constrained refinement
*S* = 1.22	*w* = 1/[σ^2^(*F*_o_^2^) + (0.0689*P*)^2^ + 0.295*P*] where *P* = (*F*_o_^2^ + 2*F*_c_^2^)/3
2640 reflections	(Δ/σ)_max_ < 0.001
223 parameters	Δρ_max_ = 0.34 e Å^−^^3^
0 restraints	Δρ_min_ = −0.28 e Å^−^^3^

### Special details

**Table:**

Geometry. All e.s.d.'s (except the e.s.d. in the dihedral angle between two l.s. planes) are estimated using the full covariance matrix. The cell e.s.d.'s are taken into account individually in the estimation of e.s.d.'s in distances, angles and torsion angles; correlations between e.s.d.'s in cell parameters are only used when they are defined by crystal symmetry. An approximate (isotropic) treatment of cell e.s.d.'s is used for estimating e.s.d.'s involving l.s. planes.
Refinement. Refinement of *F*^2^ against ALL reflections. The weighted *R*-factor *wR* and goodness of fit *S* are based on *F*^2^, conventional *R*-factors *R* are based on *F*, with *F* set to zero for negative *F*^2^. The threshold expression of *F*^2^ > σ(*F*^2^) is used only for calculating *R*-factors(gt) *etc*. and is not relevant to the choice of reflections for refinement. *R*-factors based on *F*^2^ are statistically about twice as large as those based on *F*, and *R*- factors based on ALL data will be even larger.

### Fractional atomic coordinates and isotropic or equivalent isotropic displacement parameters (Å^2^)

**Table:**

	*x*	*y*	*z*	*U*_iso_*/*U*_eq_	
C11	0.1251 (3)	0.4363 (3)	0.31823 (15)	0.0340 (5)	
C12	0.0920 (3)	0.4668 (3)	0.22728 (16)	0.0343 (5)	
H12	0.0690	0.3805	0.1926	0.041*	
C13	0.0947 (3)	0.6318 (3)	0.19058 (15)	0.0329 (5)	
C14	0.1270 (4)	0.7610 (3)	0.24223 (17)	0.0407 (6)	
H14	0.1277	0.8712	0.2161	0.049*	
C15	0.1582 (4)	0.7248 (3)	0.33299 (18)	0.0450 (6)	
H15	0.1794	0.8115	0.3680	0.054*	
C16	0.1582 (4)	0.5611 (3)	0.37241 (16)	0.0402 (6)	
H16	0.1798	0.5359	0.4333	0.048*	
N11	0.1259 (3)	0.2612 (3)	0.35946 (15)	0.0443 (5)	
N12	0.0565 (4)	0.6733 (4)	0.09503 (14)	0.0433 (5)	
O11	0.0976 (3)	0.1510 (2)	0.31125 (15)	0.0622 (6)	
O12	0.1539 (4)	0.2351 (3)	0.43994 (14)	0.0702 (6)	
H12A	0.155 (6)	0.594 (5)	0.057 (3)	0.074 (11)*	
H12B	−0.057 (6)	0.654 (5)	0.090 (2)	0.062 (10)*	
H12C	0.041 (5)	0.780 (6)	0.078 (2)	0.066 (10)*	
C21	0.6344 (3)	0.1548 (3)	0.33773 (16)	0.0390 (5)	
C22	0.6145 (3)	0.2222 (3)	0.24981 (16)	0.0359 (5)	
H22	0.6194	0.3373	0.2343	0.043*	
C23	0.5868 (3)	0.1109 (3)	0.18579 (15)	0.0346 (5)	
C24	0.5814 (4)	−0.0610 (3)	0.20828 (18)	0.0418 (6)	
H24	0.5615	−0.1333	0.1644	0.050*	
C25	0.6059 (4)	−0.1238 (3)	0.29679 (19)	0.0483 (6)	
H25	0.6046	−0.2398	0.3119	0.058*	
C26	0.6322 (4)	−0.0168 (4)	0.36326 (18)	0.0467 (6)	
H26	0.6479	−0.0586	0.4230	0.056*	
N21	0.6556 (3)	0.2741 (4)	0.40805 (15)	0.0525 (6)	
N22	0.5575 (4)	0.1777 (3)	0.09240 (14)	0.0415 (5)	
O21	0.6745 (3)	0.4197 (3)	0.38419 (14)	0.0624 (6)	
O22	0.6531 (5)	0.2192 (4)	0.48732 (15)	0.0940 (9)	
H22A	0.443 (6)	0.170 (4)	0.081 (2)	0.060 (9)*	
H22B	0.565 (5)	0.282 (6)	0.082 (2)	0.066 (10)*	
H22C	0.653 (5)	0.099 (5)	0.052 (2)	0.064 (10)*	
Cl1	0.16411 (10)	0.07512 (8)	0.06132 (4)	0.0449 (2)	
Cl2	0.66659 (10)	0.55025 (8)	0.07206 (4)	0.0456 (2)	

### Atomic displacement parameters (Å^2^)

**Table:**

	*U*^11^	*U*^22^	*U*^33^	*U*^12^	*U*^13^	*U*^23^
C11	0.0354 (11)	0.0281 (11)	0.0384 (12)	−0.0096 (9)	−0.0047 (9)	0.0038 (9)
C12	0.0376 (11)	0.0300 (11)	0.0375 (12)	−0.0115 (9)	−0.0070 (9)	−0.0025 (9)
C13	0.0366 (11)	0.0342 (12)	0.0294 (11)	−0.0105 (9)	−0.0082 (9)	0.0015 (9)
C14	0.0536 (14)	0.0324 (12)	0.0421 (13)	−0.0198 (11)	−0.0116 (10)	0.0032 (10)
C15	0.0637 (16)	0.0397 (14)	0.0403 (13)	−0.0232 (12)	−0.0165 (11)	−0.0025 (10)
C16	0.0499 (13)	0.0425 (14)	0.0311 (12)	−0.0144 (11)	−0.0124 (10)	0.0004 (10)
N11	0.0487 (12)	0.0341 (11)	0.0473 (12)	−0.0100 (9)	−0.0027 (9)	0.0072 (9)
N12	0.0573 (15)	0.0438 (14)	0.0309 (11)	−0.0155 (11)	−0.0120 (10)	0.0033 (10)
O11	0.0871 (15)	0.0324 (10)	0.0710 (14)	−0.0237 (10)	−0.0098 (11)	0.0011 (9)
O12	0.1004 (17)	0.0584 (13)	0.0515 (13)	−0.0222 (12)	−0.0179 (11)	0.0240 (10)
C21	0.0371 (12)	0.0486 (15)	0.0345 (12)	−0.0157 (10)	−0.0079 (9)	−0.0003 (10)
C22	0.0393 (12)	0.0351 (12)	0.0375 (12)	−0.0162 (10)	−0.0070 (9)	−0.0008 (10)
C23	0.0371 (11)	0.0364 (12)	0.0327 (12)	−0.0133 (9)	−0.0069 (9)	0.0005 (9)
C24	0.0460 (13)	0.0338 (13)	0.0485 (14)	−0.0153 (10)	−0.0058 (10)	−0.0041 (10)
C25	0.0528 (15)	0.0341 (13)	0.0586 (17)	−0.0151 (11)	−0.0074 (12)	0.0091 (12)
C26	0.0460 (13)	0.0542 (17)	0.0400 (13)	−0.0152 (12)	−0.0088 (10)	0.0128 (12)
N21	0.0545 (13)	0.0720 (17)	0.0382 (12)	−0.0250 (12)	−0.0122 (9)	−0.0082 (11)
N22	0.0571 (14)	0.0388 (13)	0.0344 (11)	−0.0197 (11)	−0.0118 (10)	−0.0009 (9)
O21	0.0782 (14)	0.0563 (13)	0.0612 (13)	−0.0270 (11)	−0.0155 (10)	−0.0136 (10)
O22	0.148 (3)	0.122 (2)	0.0384 (12)	−0.071 (2)	−0.0319 (13)	0.0052 (13)
Cl1	0.0625 (4)	0.0418 (4)	0.0377 (4)	−0.0221 (3)	−0.0173 (3)	0.0055 (3)
Cl2	0.0646 (4)	0.0358 (4)	0.0433 (4)	−0.0214 (3)	−0.0147 (3)	0.0000 (3)

### Geometric parameters (Å, °)

**Table:**

C11—C16	1.381 (4)	C21—C22	1.380 (4)
C11—C12	1.384 (4)	C21—C26	1.385 (4)
C11—N11	1.473 (3)	C21—N21	1.478 (4)
C12—C13	1.383 (3)	C22—C23	1.384 (3)
C12—H12	0.9300	C22—H22	0.9300
C13—C14	1.383 (3)	C23—C24	1.384 (4)
C13—N12	1.468 (3)	C23—N22	1.463 (3)
C14—C15	1.382 (4)	C24—C25	1.382 (4)
C14—H14	0.9300	C24—H24	0.9300
C15—C16	1.383 (4)	C25—C26	1.383 (4)
C15—H15	0.9300	C25—H25	0.9300
C16—H16	0.9300	C26—H26	0.9300
N11—O11	1.221 (3)	N21—O21	1.217 (3)
N11—O12	1.222 (3)	N21—O22	1.219 (3)
N12—H12A	0.93 (4)	N22—H22A	0.86 (4)
N12—H12B	0.86 (4)	N22—H22B	0.84 (4)
N12—H12C	0.85 (4)	N22—H22C	0.93 (4)
			
C16—C11—C12	123.5 (2)	C22—C21—C26	123.4 (2)
C16—C11—N11	118.2 (2)	C22—C21—N21	117.8 (2)
C12—C11—N11	118.3 (2)	C26—C21—N21	118.9 (2)
C13—C12—C11	116.7 (2)	C21—C22—C23	117.0 (2)
C13—C12—H12	121.6	C21—C22—H22	121.5
C11—C12—H12	121.6	C23—C22—H22	121.5
C12—C13—C14	121.8 (2)	C24—C23—C22	121.8 (2)
C12—C13—N12	119.2 (2)	C24—C23—N22	118.9 (2)
C14—C13—N12	118.9 (2)	C22—C23—N22	119.2 (2)
C15—C14—C13	119.3 (2)	C25—C24—C23	119.2 (2)
C15—C14—H14	120.3	C25—C24—H24	120.4
C13—C14—H14	120.3	C23—C24—H24	120.4
C14—C15—C16	120.8 (2)	C24—C25—C26	121.1 (2)
C14—C15—H15	119.6	C24—C25—H25	119.5
C16—C15—H15	119.6	C26—C25—H25	119.5
C11—C16—C15	117.8 (2)	C25—C26—C21	117.6 (2)
C11—C16—H16	121.1	C25—C26—H26	121.2
C15—C16—H16	121.1	C21—C26—H26	121.2
O11—N11—O12	123.8 (2)	O21—N21—O22	123.9 (2)
O11—N11—C11	118.0 (2)	O21—N21—C21	118.9 (2)
O12—N11—C11	118.2 (2)	O22—N21—C21	117.2 (3)
C13—N12—H12A	108 (2)	C23—N22—H22A	109 (2)
C13—N12—H12B	107 (2)	C23—N22—H22B	115 (2)
H12A—N12—H12B	107 (3)	H22A—N22—H22B	110 (3)
C13—N12—H12C	116 (2)	C23—N22—H22C	107 (2)
H12A—N12—H12C	113 (3)	H22A—N22—H22C	105 (3)
H12B—N12—H12C	104 (3)	H22B—N22—H22C	110 (3)
			
C16—C11—C12—C13	0.5 (3)	C26—C21—C22—C23	1.4 (4)
N11—C11—C12—C13	−179.22 (19)	N21—C21—C22—C23	−177.5 (2)
C11—C12—C13—C14	−0.6 (3)	C21—C22—C23—C24	−0.7 (3)
C11—C12—C13—N12	−178.8 (2)	C21—C22—C23—N22	177.8 (2)
C12—C13—C14—C15	0.3 (4)	C22—C23—C24—C25	−0.5 (4)
N12—C13—C14—C15	178.4 (2)	N22—C23—C24—C25	−179.0 (2)
C13—C14—C15—C16	0.2 (4)	C23—C24—C25—C26	1.1 (4)
C12—C11—C16—C15	−0.1 (4)	C24—C25—C26—C21	−0.4 (4)
N11—C11—C16—C15	179.7 (2)	C22—C21—C26—C25	−0.9 (4)
C14—C15—C16—C11	−0.3 (4)	N21—C21—C26—C25	178.0 (2)
C16—C11—N11—O11	−179.6 (2)	C22—C21—N21—O21	−7.2 (3)
C12—C11—N11—O11	0.1 (3)	C26—C21—N21—O21	173.9 (2)
C16—C11—N11—O12	0.7 (3)	C22—C21—N21—O22	172.9 (3)
C12—C11—N11—O12	−179.5 (2)	C26—C21—N21—O22	−6.0 (4)

### Hydrogen-bond geometry (Å, °)

**Table:**

*D*—H···*A*	*D*—H	H···*A*	*D*···*A*	*D*—H···*A*
N12—H12A···Cl2^i^	0.93 (4)	2.28 (4)	3.164 (4)	161 (3)
N12—H12B···Cl2^ii^	0.86 (4)	2.35 (4)	3.204 (4)	170 (3)
N12—H12C···Cl1^iii^	0.85 (4)	2.68 (4)	3.445 (5)	151 (3)
N12—H12C···Cl1^iv^	0.85 (4)	2.69 (4)	3.221 (3)	123 (3)
N22—H22A···Cl1	0.86 (4)	2.33 (4)	3.174 (4)	166 (3)
N22—H22B···Cl2	0.84 (4)	2.40 (4)	3.218 (4)	165 (3)
N22—H22C···Cl1^v^	0.93 (4)	2.21 (4)	3.138 (3)	170 (3)

Symmetry codes: (i) −*x*+1, −*y*+1, −*z*; (ii) *x*−1, *y*, *z*; (iii) *x*, *y*+1, *z*; (iv) −*x*, −*y*+1, −*z*; (v) −*x*+1, −*y*, −*z*.
